# Molecular epidemiology of respiratory viruses in commercial chicken flocks in Pakistan from 2014 through to 2016

**DOI:** 10.1186/s12917-019-2103-6

**Published:** 2019-10-21

**Authors:** Sajid Umar, Angélique Teillaud, Hassan Bin Aslam, Jean-Luc Guerin, Mariette F. Ducatez

**Affiliations:** 1 IHAP, Université de Toulouse, INRA, ENVT, 23 Chemin des Capelles, 31076 Toulouse, France; 2 PMAS Arid Agriculture University Rawalpindi, Rawalpindi, Pakistan; 3 University of Veterinary & Animal Sciences, Lahore, Pakistan

**Keywords:** Chicken respiratory viruses, Molecular epidemiology, Chicken, Pakistan, Avian influenza virus, Infectious bronchitis virus, Newcastle disease virus, Avian metapneumovirus

## Abstract

**Background:**

Viral diseases are a matter of great concern for poultry farmers in Pakistan. Multiple common viral respiratory diseases (CVRDs) cause huge economic losses in the poultry industry. The prevalence of CVRDs in many countries, including Pakistan, is not clearly understood.

**Results:**

Incidences of 5 chicken respiratory viruses: avian influenza virus (AIV), Newcastle disease virus (NDV/AAVV-1), infectious bronchitis virus (IBV), avian metapneumovirus (aMPV) and infectious laryngotracheitis virus (ILTV) were assessed on commercial Pakistani farms with respiratory problems from 2014 through to 2016. While AIV and AAVV-1 were frequently detected (16 to 17% of farms), IBV and aMPV were rarely detected (in 3 to 5% of farms) and ILTV was not detected. We characterized H9 AIV of the G1 lineage, genotype VII AAVV-1, GI-13 IBV, and type B aMPV strains with very little genetic variability in the 2-year study period. Co-infections with AIV and AAVV-1 were common and wild type AAVV-1 was detected despite the use of vaccines. Control measures to limit the virus burden in chicken flocks are discussed.

**Conclusions:**

Our data shows that AIV (H9), AAVV-1, IBV and aMPV are prevalent in commercial poultry in Pakistan. Further studies are necessary to assess circulating strains, economic losses caused by infections and coinfections of these pathogens, and the costs and benefits of countermeasures. Furthermore, veterinarians and farmers should be informed of the pathogens circulating in the field and hence advised on the use of vaccines.

## Background

Commercial poultry in Pakistan was established in the early 1960s, representing one of the largest agriculture-based segments of Pakistan’s economy with a significant contribution to national gross domestic product (1.3%) [1]. There are over 25,000 poultry farms spread across the country’s rural areas and the Pakistan poultry industry produces around 1220 million kg of chicken meat and 10,000 million eggs a year [2]. In the past, regional studies on poultry disease surveillance and clinical surveys have been conducted to better understand the disease distribution pattern in different regions of Pakistan [3–5]. Recently, outbreaks of viral diseases with high morbidity and mortality were being reported consistently [6–8] and are possibly due to the intensification of commercial poultry production and lack of biosecurity measures. Pakistan’s poultry industry has indeed been growing continuously, facilitating the spread of multiple common viral respiratory diseases (CVRDs) such as Newcastle disease (ND), infectious bronchitis (IB), swollen head syndrome (SHS), infectious laryngotracheitis (ILT) and low pathogenic avian influenza (LPAI) infections, which are caused by Avian Avulavirus 1 (AAVV-1), infectious bronchitis virus (IBV), avian metapneumovirus (aMPV), infectious laryngotracheitis virus (ILTV) and avian influenza virus (AIV), respectively. These are highly contagious diseases of poultry, with worldwide distribution and they have serious economic impacts on the poultry industry. The causative agents of these diseases affect chickens of all ages except ILTV, which normally does not affect chickens before 3 weeks of age. These pathogens may interact with bacterial agents resulting in high morbidity and mortality in the infected chickens [9]. The continuous emergence of new virulent genotypes from global epidemics and the frequent changes observed in the genomic sequence of these viruses lead to ineffective diagnostics and control measures. Outbreaks of CVRDs such as IB and SHS are not reported to the country’s ministry in charge of livestock and poultry production. Consequently, the distribution patterns of such chicken diseases are unclear in Pakistan [3]. Moreover, CVRDs, such as LPAI (H9N2), are of great significance to public health due to their zoonotic potential [10–12]. Therefore, it is important to investigate the distribution pattern of CVRDs in different regions and different chicken production types to develop scientific and risk-based prevention measures of these poultry diseases.

The aim of this study was to detect and characterize chicken respiratory viruses found in commercial Pakistan poultry, which is the first step of control measures implementation. Five major chicken respiratory viruses were investigated: AIV, AAVV-1, IBV, aMPV and ILTV.

## Results

### Virus prevalence and co-infections in Pakistanis farms

**Fig. 1:**
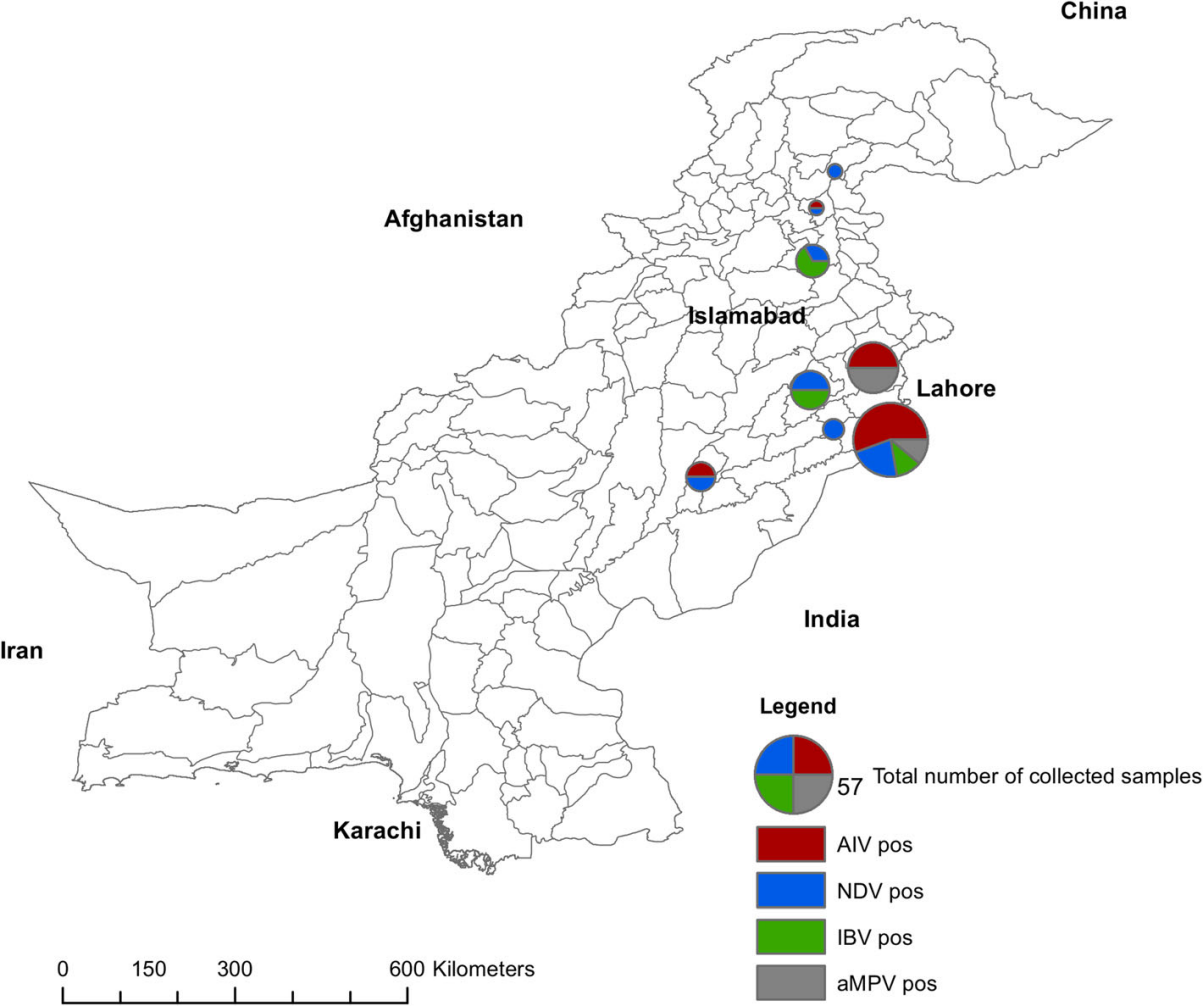
Map of Pakistan with sampling sites. Areas in Pakistan where samples were collected. The map was drawn by the authors using ArcGis and it shows the distribution of positive specimens for respective viruses (AIV in red, AAVV-1 in blue, IBV in green and aMPV in grey shades) throughout the country. The pies diameters are proportional to the number of samples collected per district

In Pakistan, samples were screened for viral respiratory pathogen nucleic acid within the commercial broiler and layer hen populations. Viral respiratory diseases are common even though vaccines are being used on a small scale in the country. In total, 161 pooled samples from 89 flocks were collected during 2014–2016 from different areas of Pakistan. Among these flocks, there were 15 (16.8%) positives for AIV (H9), 16 positives for AAVV-1 (17.9%), 4 positives for IBV (4.4%), and 2 positives for aMPV (2.2%). No ILTV positive sample was detected in our study (Fig. 1). Co-infections were common for AIV and AAVV-1 (9 AIV/ AAVV-1 co-infected flocks) but less common for the other avian viruses of interest (Table 1). Briefly, 9 flocks (6, 21, 23, 38, 41, 42, 66, 67, 68) were found positive for AIV (H9) and AAVV-1 coinfections. AAVV-1 + aMPV, AIV + aMPV and AAVV-1 + IBV coinfections were detected only in flock number 69, 70 and 80, respectively. There was no observed difference in the likelihood of co-infection between production types.

**Table 1:** Origin of the Pakistani avian samples from which viruses were sequenced

Farm ID/Flock ID	Collection date	Location	Chicken type	Flock size	Age of birds (days)	Health status	Vaccination status	Type of farm	Type of sample	Sample ID	H9	AAVV-1	IBV	aMPV	ILTV
S/6	13/7/14	Kasur	Broiler	3000	10	MRS	V (ND, IB)	open	OP	11	+	+	–	–	–
S/8	17/7/14	Rawalpindi	Broiler	10,000	16	MRS	NV	control	OP	12,15	–	+	–	–	–
H/9	19/7/14	Kasur	Broiler	3000	38	MRS	NV	open	OP	17,18	+	–	–	–	–
K/10	22/7/14	Multan	Broiler	3000	20	MRS	NV	open	OP	20	+	–	–	–	–
A/11	25/7/14	Kasur	Broiler	3000	9	MRS	NV	open	OP	21,22	+	–	–	–	–
K/12	27/7/14	Kasur	Broiler	3000	12	MRS	V (ND)	open	OP	23,24	+	–	–	–	–
R/13	29/7/14	Kasur	Broiler	2500	31	MRS	V (ND, IB)	open	OP	26		+	–	–	–
Y/21	13/8/14	Lahore	Layer	3000	80	MRS	NV	open	OP	41,42	+	+	–	–	–
A/22	14/7/14	Kasur	Layer	3000	120	MRS	NV	open	OP	44	+	–	–	–	–
R/23	15/7/14	Lahore	Broiler	10,000	31	MRS	V (ND)	control	OP	45,46	+	+	–	–	–
Z/32	23/7/14	Okara	Broiler	3000	10	MRS	NV	open	OP	64,65	–	+	–	–	–
A/38	18/10/14	Kasur	Layer	3000	78	MRS	NV	open	lung swabs	74	+	+	–	–	–
H/41	14/1/14	Multan	Layer	3000	108	MRS	NV	open	OP	77	+	+	–	–	–
H/42	28/11/14	Multan	Layer	2500	145	MRS	NV	open	OP	78	+	+	–	–	–
K/51	15/10/14	Multan	Layer	3000	66	MRS	NV	open	OP	87	–	+	–	–	–
A/59	14/8/15	Rawalpindi	Broiler	3000	14	Open mouth breathing, mucus plug in bronchi	V (ND)	open	OP	98,99	–	–	+	–	–
W/62	17/8/15	Kasur	Broiler	3000	35	Respiratory distress, congested lungs	V (ND, IBD)	open	OP, lung swabs	106,107	–	+	–	+	–
I/64	26/8/15	Rawalpindi	Broiler	3000	25	MRS	V (ND)	open	OP	110,111	–	–	+	–	–
U/65	26/8/15	Mansehra	Layer	3000	208	sickness, congested lungs, pale carcass	V (ND)	open	OP	112,113	–	+	–	–	–
T/66	29/8/15	Abbottabad	Layer	3000	180	fever, sneezing, gasping, airsacculitis	V (ND)	open	OP, lung swabs	114	+	+	–	–	–
F/67	2/9/15	Kasur	Broiler	3000	33	MRS	V (ND, IBD)	open	OP	115	+	+	–	–	–
S/70	27/9/15	Sheikhupura	Broiler	3000	35	MRS	V (IB, ND)	control	OP	122,123	+	–	–	+	–
K/80	13/12/15	Faisalabad	Broiler	3000	26	MRS	V (ND)	control	OP	142,143	–	+	+	–	–
K/84	2/1/16	Lahore	Broiler	2500	29	MRS	NV	control	OP	150,151	+	+	–	–	–
A/88	9/1/16	Kasur	Layer	3000	120	MRS	NV	open	OP	158,159	–	–	+	–	–

*V* vaccinated, *NV* non-vaccinated, *OP* oropharyngeal swab, *ND* Newcastle disease, *IB* infectious bronchitis, *IBD* infectious bursal disease, *aMPV* avian metapneumovirus, *IBV* infectious bronchitis virus, *NDV* Newcastle disease virus, *ILTV* infectious laryngotracheitis virus, *MRS* Mild respiratory signs: slight opening of the beak and chest movements. “Control” versus “open” farms: environmentally controlled (versus not controlled) poultry farms. Sample numbers: each number corresponds to a pool of 10 swabs from a chicken flock. Data in this table include only those samples and flocks which were found positive for selected viruses

### Molecular epidemiology of avian influenza virus in Pakistan

**Fig. 2:**
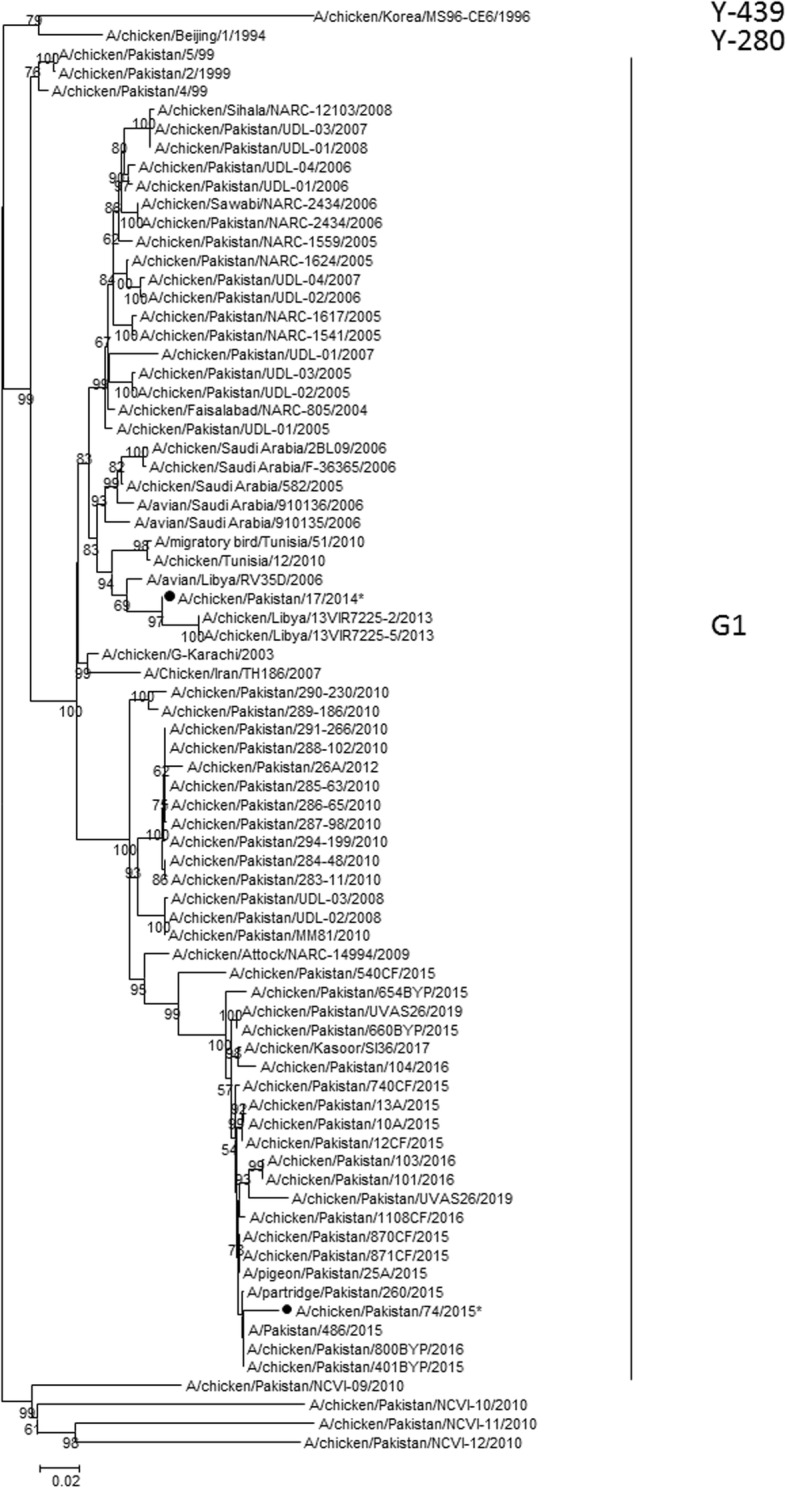
Maximum Likelihood Phylogenetic tree of recent Pakistani influenza viruses HA gene sequences. Sequences of A/chicken/Pakistan/17/2014 and A/chicken/Pakistan/74/2015 were selected as representative sequences for the present study. The HA2 nucleotide sequences of these 2 viruses are indicated in bold font with a closed circle shaped symbol. *: partial sequence

Twenty-two of the 161 collected samples were PCR positive for AIV. Partial HA sequences were obtained for 19 Pakistani strains; they were all identical but one. They all clustered with H9 influenza viruses. A/chicken/Pakistan/17/2014 and A/chicken/Pakistan/74/2015 were selected as representative sequences of 2014–2016 Pakistani samples. They cluster with G1-like viruses and are closely related to strains from Libya, Tunisia, Saudi Arabia and Pakistan, collected from 2005 through to 2015 (Fig. 2).

### Molecular epidemiology of avian avulavirus-1 in Pakistan

**Fig. 3:**
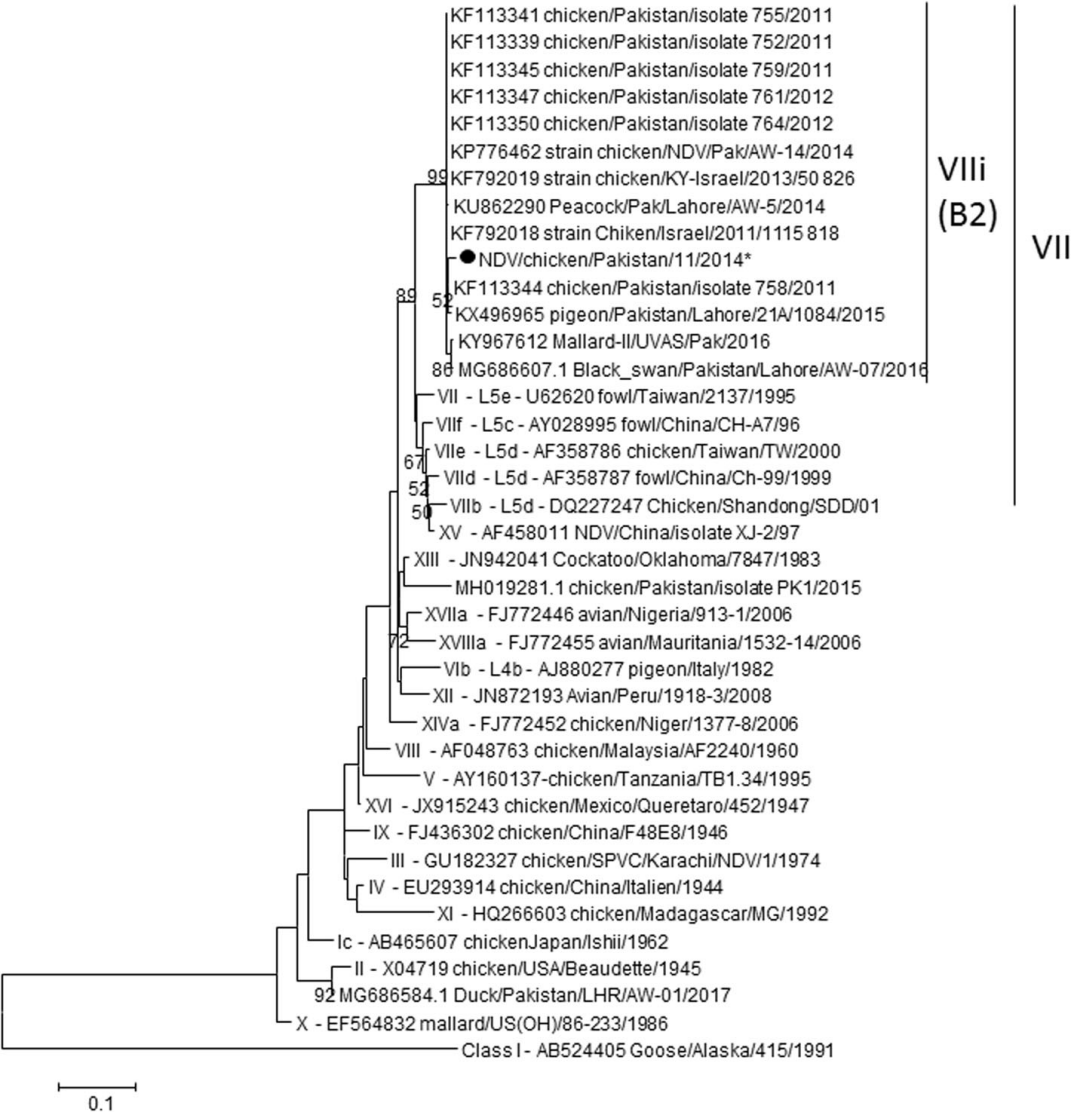
Maximum Likelihood Phylogenetic tree of recent Pakistani Newcastle disease viruses partial F gene sequences. Sequences of Pakistanis AAVV-1 were identical and AAVV-1 /chicken/Pakistan/11/2014 was selected as a representative strain for the present study. *: partial sequence

Nineteen of the 161 collected samples were AAVV-1 PCR positive. A total of 9 partial F gene sequences were phylogenetically compared with representatives of the 18 known AAVV-1 genotypes that circulate worldwide. The 9 Pakistani AAVV-1 sequences were identical; therefore, AAVV-1/chicken/Pakistan/11/2014 were used as a representative sequence for analysis. Pakistani AAVV-1 strains F protein cleavage site amino acid sequences, GRRQKR*F (aa 111–117), were indicative of high viral virulence. The phylogenetic tree clearly shows that AAVV-1/chicken/Pakistan/11/2014 clusters with sequences of genotype VII viruses (supported by a 100 bootstrap value, Fig. 3).

### Molecular epidemiology of infectious bronchitis virus in Pakistan

**Fig. 4:**
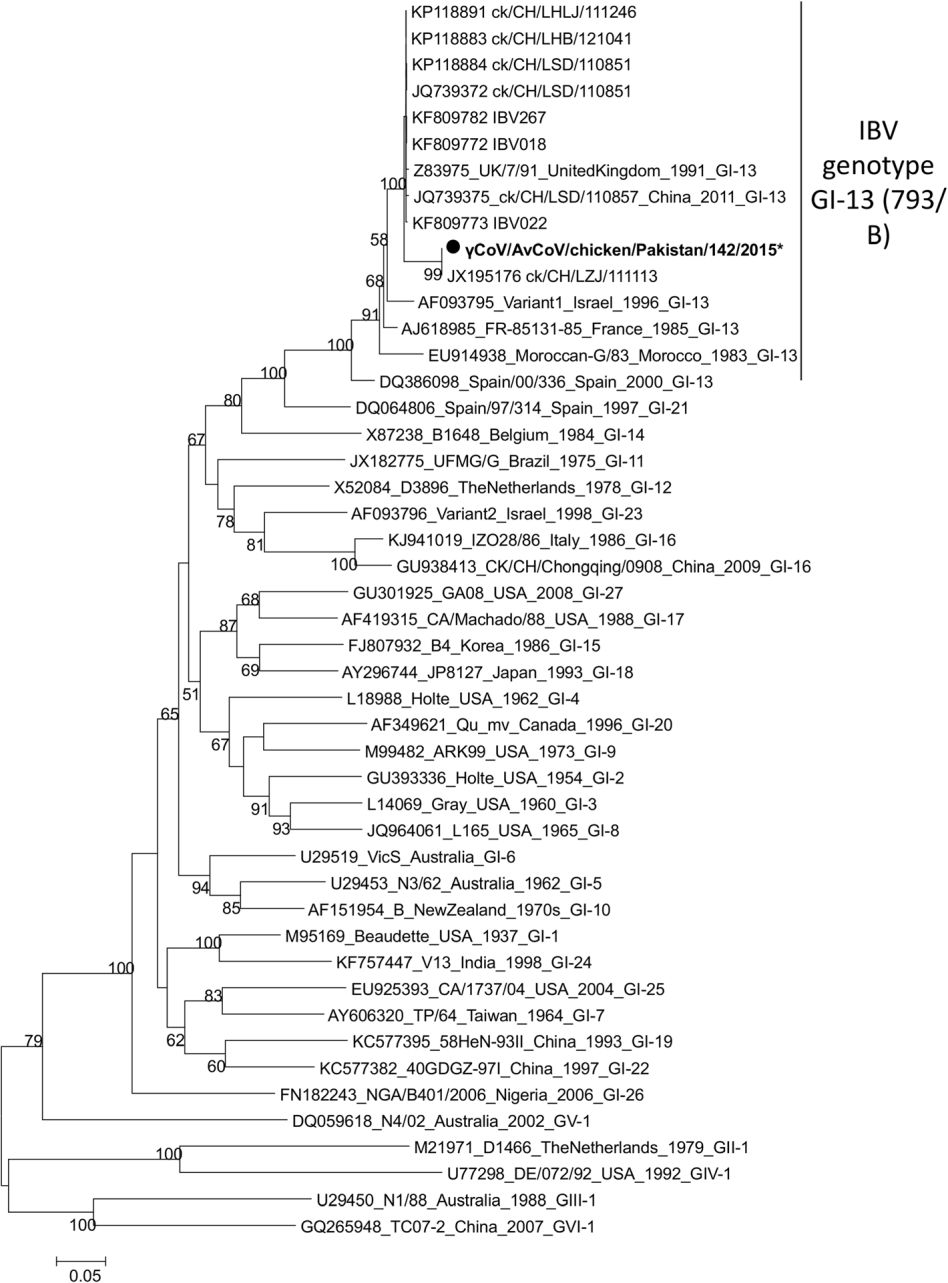
Maximum Likelihood Phylogenetic tree of recent Pakistani infectious bronchitis viruses spike gene sequences. Sequences of γCoV/AvCoV/chicken/Pakistan/11/2014, γCoV/AvCoV/chicken/Pakistan/98/2015, γCoV/AvCoV/chicken/Pakistan/99/2015, γCoV/AvCoV/chicken/Pakistan/142/2016, γCoV/AvCoV/chicken/Pakistan/143/2016, and γCoV/AvCoV/chicken/Pakistan/159/2016 were identical so only γCoV/AvCoV/chicken/Pakistan/142/2016 was represented on the tree with a black circle shaped symbol. *: partial sequence

Eight of the 161 collected samples were PCR positive for IBV. A total of 8 partial S1 gene sequences were phylogenetically compared with representatives of the 32 viral IBV genotypes circulating worldwide. As the 8 Pakistani IBV sequences were identical, γCoV/chicken/Pakistan/142/2015 was selected as a representative strain. γCoV/chicken/Pakistan/142/2015 clusters with genotype 1 lineage 13 viruses, previously called 793/B or 4/91 genotype. This is supported by a very high bootstrap value (100) (Fig. 4).

### Molecular epidemiology of avian metapneumovirus in Pakistan

**Fig. 5:**
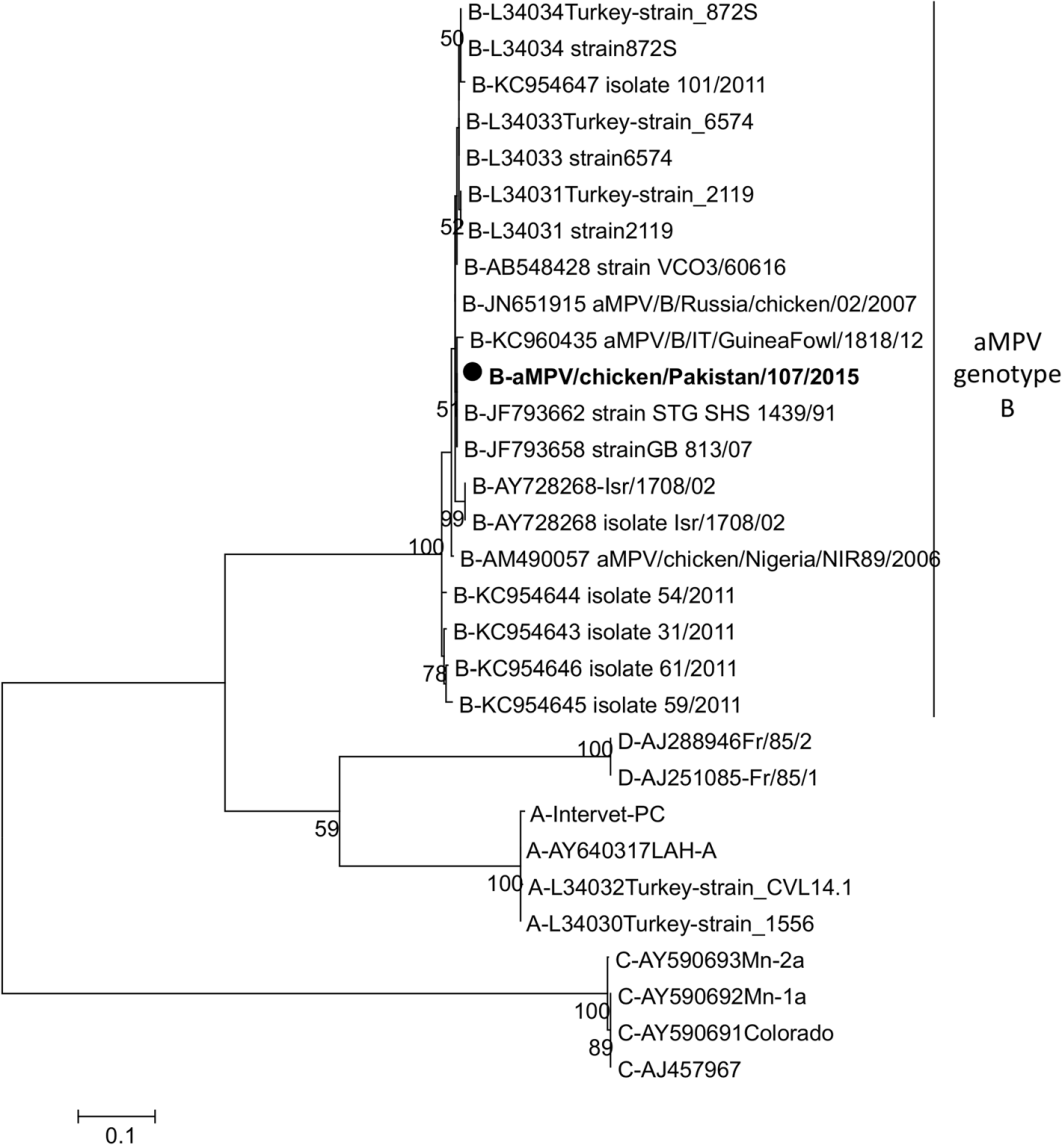
Maximum Likelihood Phylogenetic tree of recent Pakistani avian metapneumoviruses attachment protein gene sequences. Sequences of aMPV/chicken/Pakistan/106/2015, aMPV/chicken/Pakistan/107/2015, aMPV/chicken/Pakistan/122/2016, and aMPV/chicken/Pakistan/123/2016 were identical so only aMPV/chicken/Pakistan/107/2015 was represented on the tree with a black circle shaped symbol. The 4 genotypes were included here: A, B, C, and D and genotype B strains are clearly indicated on the right-hand side of the tree

Four of the 161 collected samples were aMPV PCR positive. A total of 4 partial G gene sequences were obtained, all identical, and aMPV/chicken/Pakistan/107/2015 (representative Pakistani sequence) was phylogenetically compared with representatives of the 4 known aMPV genotypes (A to D). The Pakistani sequence clusters with aMPV type B viruses (Fig. 5).

## Discussion

Viral infections of poultry cause considerable economic loss and respiratory viruses are frequently reported as primary or secondary pathogens of poultry worldwide. This work focused on the prevalence and molecular epidemiology of viral respiratory pathogens within the Pakistani commercial broiler and layer hen populations.

AIV has been frequently reported in Pakistan since 1994. LPAI H9N2, LPAI and HPAI H7N3 and HPAI H5N1 viruses have been regularly identified. Two LPAI H4N6 viruses from 2010 and 2011 have been isolated and sequenced. LPAI H9N2 and HPAI H7N3 were isolated from the same flock in 2003 [13] and since then frequent reassortment events between H7N3, H5N1 and H9N2 have been reported [10, 14]. Recently, H9N2 virus was isolated in 5.7% of 905 tested flocks of commercial and backyard poultry in Pakistan. Hemagglutinin and neuraminidase-gene-based phylogenetic analysis of commercial and backyard poultry isolates showed 100% homology within sub-lineage B2 of Pakistan [11]. The G1-like viruses detected here were closely related to previously characterized strains from Libya, Tunisia, Saudi Arabia and Pakistan. Considering the geographic proximity of these countries and the commercial exchanges, it is not surprising that these strains were detected in Pakistan.

In the present study, LPAI H9 virus was detected on several Pakistani farms from 2014 through to 2016. It would be interesting to conduct further molecular characterization to confirm that only H9 is present, link clinical signs to genes constellations, and to better understand the role of molecular determinants of field pathogenicity of currently circulating H9 viruses in unvaccinated flocks. Unfortunately, the method used to collect samples did not allow the isolation of the viruses. This resulted in limited nucleic acid quantities that did not allow for the amplification of more than the HA2 part of the genome. Similarly, only partial gene sequences were obtained for AAVV-1, IBV, and aMPV. Regardless, the circulating subtypes/genotypes of chicken viruses in Pakistan were as follows: genotype VII AAVV-1 (VIIi), GI-13 (793B) IBV and genotype B aMPV, with no difference from the summer of 2014 through to January 2016.

Avian avulavirus 1 (AAVV-1) is a member of the genus Avulavirus of the family Paramyxoviridae. The AAVV-1 species include multiple strains of avian paramyxovirus 1 (APMV-1) virus. APMV-1 is of particular importance to poultry due to virulent strains which cause Newcastle disease, often referred to as Newcastle disease viruses (AAVV-1 s). At least two classes and 18 genotypes of AAVV-1 have been described. Despite the use of imported vaccines, AAVV-1 still remains the main poultry disease in both commercial and rural chickens flocks of Pakistan [15]. Recently, AAVV-1 s of genotype VI, VII and XIII were detected in Pakistani poultry [16–23] and genotype VII is predominant across the country. Similarly, Miller et al. [24] reported that viruses of sub-genotype VIIi have replaced AAVV-1 isolates of genotype XIII in Pakistan, which were commonly isolated since 2009–2011. The viruses of genotype VII had a high genetic diversity as compared to isolates from genotypes VI and XIII and, therefore, have more potential to evolve over a period of time [25]. Partial sequences for the F gene were used for construction of phylogeny tree, which is a clear limitation of this study. Full F gene sequences would allow for a more detailed phylogeny of circulating strains of AAVV-1 in Pakistan.

Direct comparison of the IBV and aMPV findings with previous Pakistani ones is not possible due to the absence of published data. There has been so far no genomic characterization of IB viruses circulating in Pakistan, but there is serological evidence of the pathogen. Ahmed et al. [25] showed seroprevalences of IBV strains M-41 (88%), D-274 (40%), D-1466 (52%), and 4–91 (8%) in Pakistani poultry. Massachusetts strains (such as M41) are mainly used as vaccines in the field in Pakistan but we cannot totally rule out the use of 4–91 vaccines due to the limited control of imported vaccines. Similarly, high prevalence of IBV in backyard poultry (74%) and commercial poultry of Bangladesh (57%) were reported [26]. Sumi et al. [27] classified Indian IBV isolates based on phylogenetic analysis within the Mass genotype (India/LKW/56/IVRI/08, now called GI-1) and the 793/B genotype (or 4–91 genotype, India/NMK/72/IVRI/10, now called GI-13). Moreover, IBV viruses of genotype 793/B-like (GI-13) and QX-like (GI-19) have been reported recently in poultry of Iran and Iraq [28, 29]. Considering the geographic proximity of the 5 countries and the commercial exchanges, it is not surprising that 793/B (GI-13) strains were detected in Pakistan.

High seroprevalence of aMPV in broiler (48%) and breeder (93%) flocks has been reported in Iran [30]. In a recent study in Ahwaz, in the south west of Iran, Mayahi and colleagues reported 55.5% aMPV seropositivity [31]. Similarly, Eswaran et al. reported seroprevalence of aMPV in breeders (34%) of Tamil Nadu in India [32]. The virus has clearly circulated in the region. aMPV subtype B have been reported in Iran [33, 34]**.** In addition, aMPV subtype A and subtype C have been reported in China [35–37]. Again, geographic proximity and commercial exchanges of Pakistan with Iran and India rather than with China may explain the circulation of aMPV type B in Pakistan, although the PCR primers used here would not have allowed aMPV subtype C detection.

While most of the sampled farms did not vaccinate against any of the chicken viruses of interest, putative AAVV-1 vaccine failures were observed in farms 6, 13, 23, 62, 65, 66, 67, and 80 (Table 1). The AAVV-1 vaccines used in Pakistan are the LaSota (genotype II) and R2B (mesogenic Mukteswar strain, genotype III) strains. Field AAVV-1 detected in the present study (genotype VII) were therefore not vaccine viruses. The LaSota vaccine is formulated from a lentogenic (low virulence) clone of the LaSota strain and is manufactured in different countries around the world. The vaccine is imported to Pakistan and administered to commercial poultry flocks. In contrast, the R2B strain is manufactured by local vaccine companies and administered to backyard poultry. It is still unknown whether these vaccine strains (genotypes II and III viruses) can elicit a protective immune response against the prevailing field strains, especially genotype VII viruses: the protection should be evaluated in experimental and field conditions. Furthermore, the inability of live vaccines to elicit a protective immune response might be due to an inadequate cold chain supply system, inappropriate route of vaccination, or incorrect vaccination schedules. It has been reported that the currently applied AAVV-1 vaccines give better protection against the velogenic AAVV-1 s isolated from the 1930s through to the 1970s (Herts33/56, California 71) than those more recently isolated [38]. Hence parameters for selection of vaccine strains need to be reconsidered. Likewise, the optimization of vaccination schedules according to local climate and environmental conditions should be investigated.

The present study helps to understand the virus burden of Pakistani poultry production systems and highlights frequent AIV/AAVV-1 co-infections. This phenomenon has been observed previously in Bangladesh [39]. This finding may be explained by both the endemic status of the two viruses in Pakistani poultry and the poor biosecurity on farms, favoring the emergence of multiple pathogens. Experimental co-infections with the AIV and AAVV-1 showed little impact on clinical signs but altered virus shedding (with higher LPAIV than AAVV-1 shedding) [40, 41].

The main limitations of this study are (i) the type of samples collected (no virus isolation is possible from FTA cards), (ii) the thus limited amount of viral nucleic acids, and (iii) the limited knowledge available on most avian viruses circulating in the country for comparison. Further studies are warranted to fully characterize the virus strains and evaluate vaccines efficacy to counter the different pathogens.

## Conclusions

Our data showed that AIV (H9), AAVV-1, IBV and aMPV were prevalent in Pakistani commercial poultry flocks. Further studies are necessary to assess circulating strains, economic losses due to infections and coinfections, and the costs and benefits of control measures. Furthermore, veterinarians and farmers should be informed of the pathogens circulation in the field and hence advised on the use of vaccines.

## Methods

### Sample collection

From July 2014 through January 2016, 89 commercial poultry farms (broiler and layer farms) were sampled at different locations in Pakistan by participating veterinarians (Fig. 1). In total, 161 samples were collected. Sampling areas were chosen based on poultry population size: the selected sampling area is Pakistan’s main poultry producing region (with more than 50% of the country’s poultry farms, [42, 43], (Dr. Umar and Bin Aslam, personal communications). Farms were selected based on reports of clinical respiratory signs from the farmers to their veterinarians. The reported clinical signs were as follows (at least 2 signs needed to be included in the study): mortality, and/or drop in egg production, and/or drop in feed and water consumption, and/or nasal/eye discharge, and/or labored breathing, and/or gasping. Birds were sampled by trained personnel (a veterinarian with the help of the farmers who were trained on this occasion on how to swab birds) and flocks were excluded when the onset of clinical signs was observed more than 4 days prior to the visit. Farms were sampled once only (no repeated sampling). Oropharyngeal swabs or tissue impression smears (trachea, lungs) from birds showing respiratory signs were blotted onto Finders Technology Associates (FTA®) sampling cards (Whatman, Inc., Clifton, NJ). The FTA cards were allowed to air dry and were stored at 4 °C until further processing. Age, flock size and health status are summarized in Table 1. For each flock, 1 or 2 FTA cards were used, and 10 swabs were blotted on each FTA card at the farm (one sample number refers to a pool of 10 swabs from symptomatic birds in a given flock, Table 1) to detect a prevalence of ≥0.14 to ≥0.28% for each virus per flock (for flocks of 2500 to 10,000 birds with a 95% confidence level). According to the farmers’ information, some of the farms had been vaccinated against ND, infectious bursal disease and/or IB, but none of the farms visited had been vaccinated against aMPV or ILTV (Table 1).

### RNA-DNA isolation

Discs (3 mm) from FTA cards were incubated at 4 °C for 24 h in 1 mL of phosphate- buffered saline (PBS).

A comparative pilot experiment using both the QIAamp Viral RNA isolation and DNA isolation kits (Qiagen Germany) according to the instructions of the manufacturer with infectious bursal disease virus (IBDV, RNA virus) and infectious laryngotracheitis virus (ILTV, DNA virus) vaccines was performed. Real-time (RT-)PCRs were then carried out to compare the extracted material and the results were as follows: (i) extracting ILTV DNA with the QIAamp viral RNA isolation kit resulted in a loss of 1 Ct value (a twofold difference in viral DNA copies), as compared to the DNA isolated with the DNA extraction kit; (ii) extracting IBDV RNA with the Qiagen DNA extraction kit resulted in a loss of 3 Ct values (a tenfold difference in viral RNA copies), as compared to the RNA isolated with the RNA extraction kit. We therefore considered that the loss of sensitivity in detecting the DNA virus was negligible and that we could use the QIAamp Viral RNA isolation kit to extract both RNA and DNA. Viral RNA and DNA from FTA cards were therefore extracted using the QIAamp® viral RNA isolation kit (Qiagen, Germany) following the manufacturer’s instructions. RNA/DNA was eluted in 50 μL elution buffer and stored at − 80 °C until further use.

### Reverse transcriptions (RT) and polymerase chain reactions (PCR)

Complementary DNA (cDNA) was generated for RNA viruses from 5 μL of extracted RNA using RevertAid first strand cDNA synthesis kit (RevertAid First Strand cDNA Synthesis kit, ThermoFisher Scientific, Carlsbad, CA) following the manufacturer’s protocol. Briefly, 5 μL of total RNA was mixed with random hexamer primers (0.3 μg/μL) and incubated for 5 min at 65 °C. Then, 4 μL of 5x reaction buffer (ThermoFisher Scientific, Carlsbad, CA), 0.5 μL of RNase Out (Life Technologies, Carlsbad, CA), 2 μL of 10 mmol/L deoxynucleoside triphosphate (dNTP) solution (Finnzymes, Espoo, Finland), and 1 μL of RevertAid reverse transcriptase (ThermoFisher Scientific, Carlsbad, CA) were added. Water was added to reach a final reaction volume of 20 μL. The RT reaction was completed following 10 min at 25 °C then 60 min at 42 °C, and finally 10 min at 70 °C. cDNA was used as the template for PCR amplification. The primers and PCR conditions used for the detection of AAVV-1, IBV, aMPV (types A and B), ILTV, and AIV are listed in Table 2. Two different Taq DNA Polymerases were used in this study (Qiagen Taq DNA polymerase, Germany, for the 2014 samples; and Kapa biosystems, Inc. MA, for the 2015–2016 samples). All PCR programs were performed with a GeneAmp PCR system 9700 thermal cycler (Applied Biosystems, USA). PCR amplicons were analysed using 1.5% agarose gels (Ultrapure, Invitrogen, Merelbeke, Belgium), containing nucleic acid stain (SYBR® Safe DNA gel stain, ThermoFisher Scientific, Carlsbad, CA) and using 1 × TBE as electrophoresis running buffer. Bands sizes were compared to a commercially available 100 bp ladder (Bioline HyperLadder™ 100 bp). Attenuated live vaccine viruses: Newcastle Disease Vaccine B1 Type LaSota Strain (Merial) for AAVV-1, MassH120 (Bioral H120) for IBV, Nobilis TRT (MSD) for aMPV, and A/turkey/Italy/977/99(H7N1) for AIV were used as positive controls. Negative (RNAse/DNAse free H_2_O) controls were included in each reaction.

**Table 2:** PCR conditions for the detection and genotyping of avian respiratory viruses in samples from Pakistan

Virus		Primers	Sequences (5–3)	Target	Amplicon size (bp)	PCR conditions	Reference
Detection PCR	AAVV-1	FIP-1	5′ TACTTTGCTCACCCCCCTT 3’	Fusion gene (F)	280	94 C for 2 min; 40 cycles of 94 C for 30 s, 58 C for 30 s, 72 C for 1 min; final extension at 72 C for 5 min	[48]
		FIP-2	5’ CATCTTCCCAACTGCCACT 3’				
	IBV	N791	5’ GTGATGACAAGATGAATGAGGA 3’	Nucleo-protein gene (N)	380	94 C for 2 min; 40 cycles of 94 C for 30 s, 54 C for 30 s, 72 C for 1 min; final extension at 72 C for 5 min	[49]
		N1129	5’ CAGCTGAGGTCAATGCTTTATC 3’				
	ILTV	gEU gEL	5’ GCTGGGTTCTGGGCTACACAAC 3′ 5′ TGCGCGTGACTCGGAGAG 3’	Glyco-protein E gene (gE)	626	94 C for 2 min; 40 cycles of 94 C for 30 s, 61 C for 30 s, 72 C for 1 min; final extension at 72 C for 5 min	[50]
	aMPV	G1^a^	5′ GGGACAAGTATCYMKAT 3’	Attachment glycoprotein gene (G)	441	94 C for 2 min; 40 cycles of 94 C for 30 s, 50 C for 30 s, 72 C for 1 min; final extension at 72 C for 5 min	[51]
		G6^a^	5′ CTGACAAATTGGTCCTGATT 3’				
	AIV	M52C	5′ CTTCTAACCGAGGTCGAAAG 3’	Matrix gene (M)	280	95 C for 30 s; 40 cycles of 95 C for 30 s, 55 C for 30 s, 72 C for 30s; final extension at 72 C for 1 min	[52]
		M253R	5’AGGGCATTTTGGACAAAKCGTCTA 3’				
Genotyping PCR	AIV	HA-1134F	5′ GGAATGATHGAYGGNTGGTATG 3′	hemma-gglutinin gene (HA)	600	95 C for 30 s; 40 cycles of 95 C for 30 s, 55 C for 30 s, 72 C for 30s; final extension at 72 C for 1 min	[53]
		NS-890 R	5′ ATATCGTCTCGTATTAGTAGAAAC AAGG 3′				
	IBV	S15	5′ TGAAAACTGAACAAAAGACA 3’	Spike gene (S)	700	95 °C for 2 min; 40 cycles of 95 °C for 30 s, 52 °C for 30 s, 72 °C for 30 s; final extension of 72 °C for 12 min	[54]
		CK2	5′ CTCGAATTCCNGTRTTRTAYTGRCA 3′				

*bp* base pairs

^a^this primer pair does not detect serotype C viruses

### Sequencing

PCR products were purified using the NucleoSpin®Gel and PCR Clean-up kit (Macherey-Nagel, Düren, Germany) following the manufacturer’s instructions. Purified products were quantified with a Qubit® 2.0 fluorometer (ThermoFisher Scientific, Waltham, CA). The PCR primers and 10 ng of DNA were used for sequencing in both directions using a Big Dye Terminator v.3.1 cycle sequencing kit (Applied Bio-systems) on a capillary sequencer (model 3100, Applied Bio-systems). In case of nucleotide ambiguity, sequencing was repeated. Sequences generated in the present study were submitted to the EMBL/GenBank database under the accession numbers LT599493 to LT599497.

### Data analysis and phylogeny

Assembly and analysis of sequence data were conducted using the BioEdit Software version 5.0.9 [44]. Additionally, the software was used to read the sequencing electrophoregrams and to exclude nucleotide ambiguity. To ensure the reliability of sequences, forward and reverse sequences were aligned with ClustalW [45]. Phylogenetic analyses were performed using the maximum likelihood method, the Tamura-Nei model (gamma distributed), with 500 bootstrap replicates and pairwise deletion using MEGA software Version 6.06 [46]. For reference, bootstrap values above 50 were labelled on major tree branches. The nucleotide sequences of partial segments of the S1, G, HA, and F genes of IBV, aMPV, AIV and AAVV-1, respectively, were compared with the first 10 blast hit sequences and reference sequences of the same genes from GenBank. Recent classifications for IBV genotypes were used [47].

## Data Availability

The datasets generated and/or analysed during the current study are not fully publicly available due to a confidentiality agreement on farms and farmers exact identification/location but raw laboratory datasets are available from the corresponding author on reasonable request.

## Abbreviations

AAVV-1: Avian Avulavirus/Newcastle disease virus
AIV: Avian influenza virus
aMPV: Avian metapneumovirus
APMV-1: Avian paramyxovirus 1
cDNA: Complementary DNA
CVRD: Common viral respiratory diseases
DNA: Deoxyribonucleic acid
dNTPs: Deoxynucleoside triphosphate
HA: Haemagglutination
IB: Infectious bronchitis
IBV: Infectious bronchitis virus
ILTV: Infectious laryngotracheitis virus
ND: Newcastle disease
OP: Oropharyngeal
PBS: Phosphate buffer saline
PCR: Polymerase chain reaction
RNA: Ribonucleic acid
SHS: Swollen head syndrome
